# Efficacy of selective laser trabeculoplasty for normal tension glaucoma: 1 year results

**DOI:** 10.1186/1471-2415-15-1

**Published:** 2015-01-08

**Authors:** Jacky WY Lee, Wing Lau Ho, Jonathan CH Chan, Jimmy SM Lai

**Affiliations:** Department of Ophthalmology, Caritas Medical Centre, 111 Wing Hong Street, Kowloon, Hong Kong; Department of Ophthalmology, The University of Hong Kong, Kowloon, Hong Kong; Department of Ophthalmology, Queen Mary Hospital, Kowloon, Hong Kong

**Keywords:** Selective laser trabeculoplasty, Normal tension glaucoma, Intraocular pressure, Medication

## Abstract

**Background:**

Normal tension glaucoma (NTG) is commonly treated with anti-glaucoma medications. Recently, selective laser trabeculoplasty (SLT) has been demonstrated to lower the intraocular pressure (IOP) and medication use in NTG. The purpose of this study was to investigate the efficacy of a single session of SLT for NTG at 1 year.

**Methods:**

This prospective cohort study recruited NTG patients taking anti-glaucoma medication. Potential subjects were excluded if they had had previous glaucoma surgery or laser and also if intraocular surgery or additional SLT procedures were performed after the first treatment. All subjects underwent a 1-month washout. A 30% IOP reduction was set as the target IOP. A single session of SLT was performed to 360 degrees of the trabecular meshwork. At 1-month after SLT, medication was resumed to achieve the target IOP. The IOP was measured every 3 months, and the number of medications was recorded at 3, 6, and 12 months. Only the right eye was used for statistical analysis.

**Results:**

In 41 right eyes, the mean pre-study IOP was 14.3 ± 3.4 mmHg while on 1.5 ± 0.8 eye drops. The post-washout IOP was 16.2 ± 2.2 mmHg. A mean of 191.1 ± 26.3 SLT shots at 1.0 ± 0.07 mJ were applied. There was significant IOP reduction at all time intervals following SLT when compared to the post-washout IOP (P < 0.0001). The number of medications was significantly reduced at all time intervals following SLT when compared to the pre-study level (P < 0.0001). At 12 months, the mean IOP was 12.2 ± 2.2 mmHg while on 1.1 ± 0.9 eye drops.

**Conclusions:**

A single session of SLT for NTG achieved an additional 15% IOP reduction while using 27% less medication at 1 year compared to pre-study levels.

**Trial registration:**

The Clinical Trials Register of the University of Hong Kong HKCTR1847

The European Clinical Trials Database 2014-003305-15 (August 11, 2014) (https://www.clinicaltrialsregister.eu/ctr-search/search?query=2014-003305-15)

## Background

Normal tension glaucoma (NTG) accounts for the majority of primary open angle glaucoma (POAG) in Asian countries like Japan and Korea [1, 2]. Selective laser trabeculoplasty (SLT) is a well-established treatment for POAG with a comparable efficacy to medications and its high safety profile and repeatability being its greatest strengths [3–9]. However, evidence on the use of SLT for NTG is scanty in the literature, with reported IOP reductions ranging from 12 to 15% in only a few small case series involving 11 to 18 subjects [10, 11]. Recently, the 6-month results on SLT treatment in 46 NTG subjects demonstrated a 20% IOP reduction in addition to 27% fewer anti-glaucoma medications compared to pre-study levels [12]. The effect of SLT is known to wear off with time [4]. The objective of this study was to investigate the sustainability of SLT’s efficacy in treating NTG at 1 year following laser.

## Methods

This study adhered to the tenets of the Declaration of Helsinki. Informed patient consent and approval by the Institutional Review Board of The Hospital Authority of Hong Kong were obtained prior to study commencement. The authors declare no financial or conflicting interests.

The study was registered with the following publicly accessible registries: the Clinical Trials Register of the University of Hong Kong (trial registration number: HKCTR1847) in April 2012 and retrospectively registered with the European Clinical Trials Database (trial registration number: 2014-003305-15) on August 11, 2014.

The methodology of this study has been previously described in parts [10]. This was a prospective cohort study from July 2012 to March 2014, conducted at a university hospital in Hong Kong.

The study recruited cases of unilateral or bilateral NTG subjects who were currently on topical anti-glaucoma medications. NTG was defined by open angle on gonioscopy, glaucomatous visual field loss on Humphrey visual field analyzer as per the Hodapp-Parrish-Anderson’s criteria [13], progressive thinning of the retinal nerve fiber layer on Optical Coherence Tomography, and Goldmann applanation measured IOP < 21 mmHg on all documented clinical visits. Cases were excluded if they had received prior surgery or laser for the treatment of glaucoma or taking any systemic medications that may affect IOP. Patients were also excluded if there were contraindications for SLT like corneal pathologies or scars; they did not complete follow-up visits to12 months, or if any intraocular surgery or repeated SLT was performed within 12 months of the first SLT treatment.

The pre-study IOP with anti-glaucoma medication and the number of anti-glaucoma medication used were recorded prior to study enrollment. Fixed combination eye drops were counted as two types of anti-glaucoma medication. All patients then underwent a 1-month washout period where all anti-glaucoma medications were discontinued. A mean baseline IOP without medication was then calculated after IOP phasing at 9 am, 1 pm, and 5 pm. An individual target IOP was calculated as a 30% reduction from the baseline IOP, as per the findings from The Collaborative Normal Tension Glaucoma Study [14].

All patients received a single session of SLT using a Q-switched Nd:YAG laser (Ellex Solo™, Ellex Medical Pty. Ltd., 82 Gilbert Street, Adelaide, SA 5000 Australia) with an initial energy of 0.8 mJ. The power was titrated up or down until bubble formation was just visible. A single glaucoma specialist (JWYL) delivered the SLT treatment and both eyes were treated in the same laser session for those with bilateral disease. In all treated eyes, a single drop of Brimonidine Tartrate (Alphagan P, Allergan Inc., Waco Texas, United States of America) was instilled immediately after SLT. Dexamethasone 0.1% and Neomycin 0.5% combination eye drop (Dexoptic-N by Ashford Laboratories Pvt. Ltd., 31/36, 5th Floor, Dheeraj Heritage,S. V. Road, Santacruz West, Mumbai - 400 054, India) was used twice daily for 1 day and was continued for a few more days only if anterior chamber reaction was seen on day 1 after SLT. Subjects returned for follow-up on day 1, 1 week, 1 month, 3 months, 6 months, 9 months, and 12 months after SLT. At 1 month after SLT, IOP phasing (9 am, 1 pm, and 5 pm) was repeated and a mean 1-month IOP was calculated. IOP phasing was only performed before and at 1-month after SLT. Anti-glaucoma medications were resumed and titrated based on clinical response to achieve the preset target IOP for each individual. The order of resuming anti-glaucoma medication included the following: first, alpha-adrenergic agonists or prostaglandin analogues were prescribed followed by topical carbonic anhydrase inhibitors and then lastly, β-blockers. This order was based on the understanding from the Low-pressure Glaucoma Treatment Study that NTG subjects treated with alpha-adrenergic agonists were less likely (9.1%) to develop visual field progression than those using β-blockers (39.2%) [15]. When multiple medications were required, fixed combination medications were given to simplify the drug regimen.

The primary outcome measure included IOP at the following time intervals: pre-study with medication, baseline phasing after washout, 1 day, 1 week, 1 month post-SLT phasing, without medication, 3 months, 6 months, 9 months, and 12 months after SLT.

The secondary outcomes included these: the number of anti-glaucoma medications used pre-study and again at 3 months, 6 months, and 12 months after SLT. Goldmann applanation tonometry was used to measure IOP.

### Definitions of success

- Absolute success: IOP reduction ≥ 20% after SLT compared to baseline without any additional anti-glaucoma medication
- Qualified success: IOP reduction ≥ 20% reduction compared to baseline, with additional anti-glaucoma medication

### Statistics

Only the right eye was used for statistical analysis. The Friedman test with Dunn’s Multiple Comparison posthoc test was used to calculate the following outcome measures over the study period:

1. IOP at pre-study and at 1 day, 1 week, 1 month, 3 months, 6 months, 9 months, and 12 months post-SLT.
2. Number of anti-glaucoma eye drops at pre-study and 1 month, 3 months, 6 months, and 12 months post-SLT.

A Kaplan-Meier survival curve was used to represent the “mortality” after SLT, which was defined as the need of a repeated SLT procedure during the study period.

All means were expressed as mean ± standard deviation. Statistical significance was defined as P < 0.05.

## Results

In the initially recruited 46 NTG subjects, one was deceased prior to the 12-month follow-up, two needed a secondary SLT treatment within the first 12 months, and two had a phacoemulsification with intraocular lens insertion. In the remaining 41 subjects, the mean age was 64.7 ± 11.9 years and with 21 male and 20 female subjects. There were 41 right eyes and 33 left eyes. All subjects were ethnic Chinese with pigmented and open angle configurations.

The 41 right eyes were used for statistical analyses. The mean pre-study IOP was 14.3 ± 3.4 mmHg while on 1.5 ± 0.8 anti-glaucoma eye drops. The mean baseline IOP after washout of anti-glaucoma medication was 16.2 ± 2.2 mmHg. The central corneal thickness was 550 ± 36.9 micrometres. The mean SLT shots applied was 191.1 ± 26.3 per session per eye using a mean energy of 1.0 ± 0.07 mJ. There were no complications from SLT.

**Figure 1:**
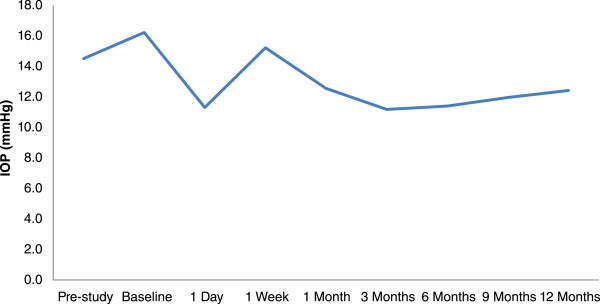
**Changes in IOP before and after SLT for NTG.**

When using the pre-study IOP for comparison, there was significant IOP reduction at all time intervals following SLT (all P < 0.0001) except at 1 week, where there was no significant difference with the pre-study IOP (P > 0.05). The 1-week IOP was significantly higher than the other post-SLT time intervals (1 day, 1 month, 3-month, 9-month, and 12-month) (all P < 0.0001). There was no significant difference between the 1-month IOP versus the other post-SLT time intervals (all P > 0.05). (Table 1 and Figure 1).

**Table 1:** **IOP and number of anti-glaucoma eye drops before and after SLT for NTG**

	Pre-study	Base-line	1 Day	1 Week	1 Month	3 Months	6 Months	9 Months	12 Months
**Mean IOP (mmHg)**	14.3 ± 3.4	16.2 ± 2.2	11.2 ± 2.3	14.7 ± 3.1	12.4 ± 2.0	11.2 ± 1.9	11.3 ± 1.6	11.7 ± 1.9	12.2 ± 2.2
**Mean number of anti-glaucoma eye drops**	1.5 ± 0.8	0	0	0	0.9 ± 0.9	-	1.0 ± 1.0	-	1.1 ± 0.9

**Figure 2:**
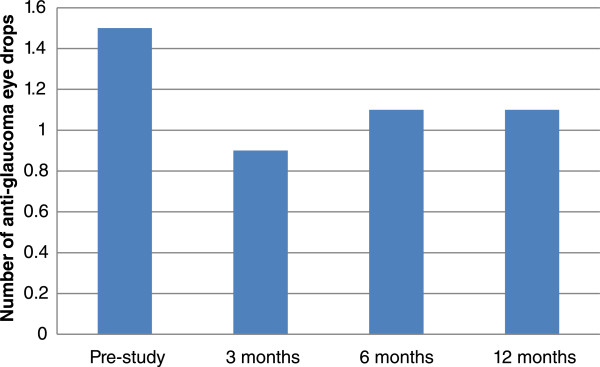
**Changes in the number of anti-glaucoma eye drops before and after SLT for NTG.**

The number of anti-glaucoma medications was significantly reduced at all time intervals (3, 6, and 12 months) following SLT when compared to the pre-SLT level (all P < 0.0001). There was no significant difference between the numbers of medication at 3, 6, or 12 months after SLT (all P > 0.05) (Table 1 and Figure 2).

**Figure 3:**
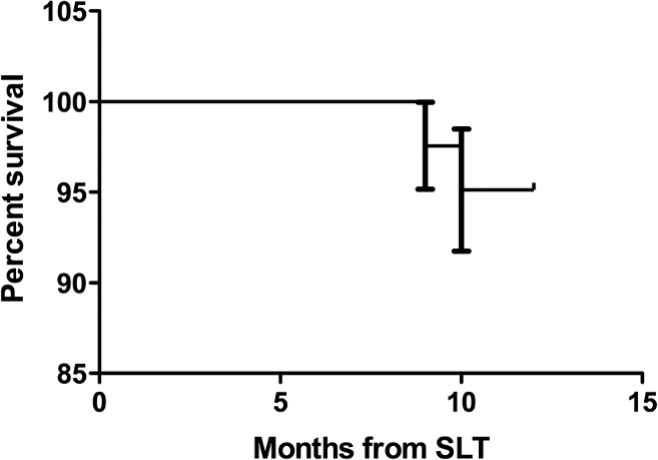
**Kaplan-Meier survival curve following SLT, where mortality = need of repeated procedure SLT during the study period.**

At 12 months post-SLT the mean IOP was 12.2 ± 2.2 mmHg while on 1.1 ± 0.9 anti-glaucoma eye drops. This represented a 14.7% reduction in IOP in addition to a 26.7% reduction in anti-glaucoma eye drops compared to pre-study levels. Absolute success was achieved in 22.0% (9/41), and qualified success was achieved in 73.2% (30/41) at 12 months following SLT. During the study period, the mean survival rate of the procedure was 95.1%; only two subjects required a repeated SLT with 12 months of the first procedure (Figure 3).

## Discussion

As early as the 1980’s, Argon Laser Trabeculoplasty (ALT) was used to successfully treat NTG, achieving IOP reductions of 4.9 mmHg at 12 months with a gradual tapering of the pressure-lowering effect over the course of a 21.6-month follow-up [16]. The trabecular scarring and destructive nature of ALT limited its clinical practicality for those who lost the pressure-lowering effective with time. Understanding the sustanability of SLT is important because unlike ALT, SLT employs a low energy, nanopulse technology with minimal coagulation damage to the trabecular meshwork and cornea, so the laser can be repeated for those who show an initial response that is gradually lost over time [17, 18].

There was a significant IOP spike at 1 week following SLT when compared to the other post-SLT time intervals, but there was no significant difference between the 1-week and pre-SLT IOP. This was followed by a gradual reduction in IOP and a plateau from 3 to 12 months. The IOP was reduced from 12.4 ± 2.0 mmHg at 1-month to 11.2 ± 1.9 mmHg at 3 months after the resumption of anti-glaucoma medication in subjects who fell short of reaching their target IOP. We did notice a gradual decline in the absolute success of 61% at 6 months [10] to an absolute success of 22% at 12-months. This entailed that a proportion of subjects did required the resumption of anti-glaucoma medications, as the effect of SLT is well known to decrease with time. However, there was no significant difference in the number of medications between 3 to 12 months (P > 0.05). Thus, these findings suggest that the effectiveness of SLT was quite consistent and preserved for up to 1 year after laser.

To the best of our knowledge, this is one of the larger longitudinal studies reporting the effects of SLT in NTG. Our research demonstrated that at 1 year, treated eyes achieved a 15% IOP reduction while using 27% less medication compared to the pre-study levels. Our findings were consistent with that of Nitta et al. who similarly reported a 1 year IOP reduction of 16.5% in NTG subjects who received SLT as primary treatment [19]. Similarly, in studies that had a mixed population of POAG, NTG, and ocular hypertension subjects, the 1-year IOP reduction ranged from 14 to 15% [10, 20]. In a retrospective review involving 18 NTG subjects, there was no significant difference between the number of medications pre-SLT (1.68 ± 1.11) and post-SLT (1.45 ± 1.18) (P = 0.178) at a mean of 9.9 months follow-up [11]. This difference to our findings could be attributed to the fact that the degree of SLT treatment in the retrospective study ranged from 180 to 360 degrees [11] while in our study, all patients received 360 degrees of treatment. This has been associated in the literature with better results than 180 degrees treatment in POAG subjects [21].

The degree of IOP reduction in NTG (15% at 1 year in our study) is not as dramatic as in POAG or even primary angle closure glaucoma (PACG), which has been reported to be around 32% at 5 years and 24% at 6 months respectively by Lai et al. for a similar Chinese population [22, 23]. This is probably attributed to the lower pre-treatment IOP in NTG, as a higher pre-treatment IOP is by far one of the most consistent predictors of SLT success [5, 24–26]. The pre-treatment IOPs in the above-mentioned studies were 26.8 mmHg, 24.6 mmHg, and 16.2 mmHg for the POAG, PACG, and NTG groups respectively.

Our study had its limitations. Firstly, it would have been ideal not to use adjuvant anti-glaucoma medication at all to observe the IOP-lowering effect of SLT alone. However, this would mean that the subjects would be at a suboptimal IOP and put them at an unnecessary risk of disease progression. On the other hand, comparing IOP and the number of medications before and after SLT is more representative of real life clinical situations where SLT is often added as adjuvant therapy for those who are already on anti-glaucoma medications, rather than the primary and only treatment for NTG. Secondly, a number of subjects were intolerant to anti-glaucoma medications or refused additional medication after SLT. Therefore, not everyone was able to achieve the ideal 30% IOP reduction as per the Collaborative Normal Tension Glaucoma [14]. Thirdly, providing a 24-hour IOP monitoring in future studies would enlighten us on the circadian efficacy of SLT as circadian IOP fluctuations has been demonstrated to be clinical important for glaucoma patients [27].

## Conclusions

At 1 year after a single session of SLT for NTG, the IOP was lowered by 15% and with 27% fewer eye drops than the pre-study level. The absolute success rate was 22% and the qualified success was 73%.

## Abbreviations

NTG: Normal tension glaucoma
SLT: Selective laser trabeculoplasty
IOP: Intraocular pressure.
